# *OX40* Gene and Serum Protein Expression Profiles in Patients with Parkinson’s Disease

**DOI:** 10.22074/cellj.2018.5038

**Published:** 2018-03-18

**Authors:** Azadeh Seyedjoodaki, Fereshteh Alsahebfosoul, Nahid Eskandari, Vahid Shaygannejad, Mansour Salehi, Mohammad Kazemi, Mostafa Manian, Omid Mirmosayyeb, Mohammad Taghi Kardi

**Affiliations:** 1 Department of Immunology, School of Medicine, Isfahan University of Medical Sciences, Isfahan, Iran; 2 Applied Physiology Research Center, School of Medicine, Isfahan University of Medical Sciences, Isfahan, Iran; 3 Department Neuroscience, School of Medicine, Isfahan University of Medical Science, Isfahan, Iran; 4 Department of Genetics and Molecular Biology, School of Medicine, Isfahan University of Medical Science, Isfahan, Iran; 5 Department of Immunology, School of Medicine, Iran University of Medical Sciences, Tehran, Iran; 6 Department of Biology, Faculty of Science, University of Isfahan, Isfahan, Iran

**Keywords:** Neurodegenerative, *OX40*, Parkinson’s Disease

## Abstract

**Objective:**

Inflammation of the immune system and the central nervous system has been known as an important predisposing factor for Parkinson’s disease (PD). Increased expression of *OX40* protein (CD134) is a known factor for increased inflammation and initiation of NF-kappa-B signaling pathway in different diseases. We aimed to investigate the expression of *OX40* at the transcript and serum protein levels.

**Materials and Methods:**

Twenty individuals with PD and 20 healthy individuals, as controls, were enrolled in this casecontrol study. Expression of *OX40* at the transcript level and serum protein levels were measured by quantitative real-time polymerase chain reaction (qRT-PCR) and enzyme-linked immunosorbent assays respectively.

**Results:**

The mean expression level of *OX40* was increased in patients but not at a significant level (P>0.05). Consistently, the mean serum concentration of *OX40* showed a mild, but non-significant, increase in the patients (P>0.05).

**Conclusion:**

We conclude that *OX40* expression at either the transcript or protein level has no diagnostic utility in asymptomatic PD. This shows the need for clinical, cellular and interventional research to detect new robust biomarkers.

## Introduction

Parkinson’s disease (PD) is the second leading neurodegenerative disease after Alzheimer’s disease, which causes symptoms such as slowness of movement or vibration in the hands and other limbs (1). PD mainly affects the central nervous system (CNS) and consequently the motor system. This disease generally begins with death and reduction of dopaminergic neurons in substantia nigra and progresses with the accumulation of Lewy bodies, comprising alpha-synuclein and ubiquitin (2). Tremor of hands, muscle stiffness in movement and slow movements during walking are some of the early clinical symptoms of PD. Following the incidence of early symptoms and the progression of the disease, behavioral and emotional problems become more apparent including dementia, depression, sleep problems and intellectual disability (3).

Several studies have led to the identification of a number of genes implicated in PD pathogenesis and have therefore, to some extent, shed light on the pathogenic mechanisms of this disease. Alphasynuclein (autosomal dominant), Lewy body-positivePARK1 and PARKIN (autosomal recessive), juvenilePARK2 and Lewy body-negative are some of thesegenes (4, 5). In addition, many studies have beenconducted at the protein level, profiling cells involvedin PD. These studies have indicated that the expression rate of proteins in different molecular pathways including glycolysis, tricarboxylic acid, apoptoticpathways, and cell viability as well as cytoskeletonrelated proteins change significantly, thus being associated with the pathogenesis of PD (6, 7).

The interaction effect between inflammation and neuron dysfunction is complicated and the causes of PD have not yet been elucidated. However, there is ample evidence for dysfunction and inflammation of the immune system and CNS as important predisposing factors for the development of PD (8, 9). Activation of necroptosis pathways in neurons may cause activation of immune-inflammatory pathways and by producing neurotoxic cytokines may lead to neuronal death (10). Suppression of inflammation responses in innate and acquired immunity and changes in the expression rate of proteins involved in this process, as a basis for a highly efficient treatment, have attracted much attention in the recent years.

A key gene involved in immune-inflammatory pathways, *OX40*, encodes the protein *OX40* (Kd50), also known as CD134 and TNFRSF4 and functionally known as a tumor necrosis factor receptor. This receptor helps activate the NF-kappa-B pathway through binding to the adaptor proteins TRAF2 and TRAF5 (11). Moreover, activation of T cells, using CD134L-expressing cells, results in clonal expansion, and production and secretion of cytokines. There is evidence showing that *OX40* plays a part in inducing T cell responses in autoimmune diseases, and blocking *OX40* signaling may be considered as a treatment approach for many autoimmune diseases. 

The direct correlation between OX40 protein level in peripheral blood, and severity and progression of autoimmune diseases has been studied (12-14). Changes in OX40 protein expression has also been investigated in certain diseases such as atherosclerosis, rheumatoid arthritis, and inflammatory diseases of bowel and muscle (15-17). Moreover, the effect of suppressing the interaction between *OX40* and its receptor has been investigated for the treatment of many diseases including asthma and allergy, atherosclerosis, diabetes, and systemic lupus erythematosus (SLE) (18-20). 

Given that the *OX40* expression level in the peripheral blood of people with PD may provide insight into the immunologic mechanisms related to PD progression, we aimed to examine the association of *OX40* expression with PD for potential diagnosis of PD and to develop more effective treatment approaches.

## Materials and Methods

In this case-control study, patients with PD who were referred to Kashani Hospital of Isfahan volunteered to participate were enrolled if diagnosis of PD was confirmed by magnetic resonance imaging (MRI) or by a specialist according to the clinical symptoms including tremors or shaking, stiffness and muscle pain, limited and slow movements, pain and balance disorders (UK Parkinson’s Disease Society Brain Bank Clinical Diagnostic Criteria) (21). Before enrollment, adequate explanations were given to the individuals, and written informed consent were obtained. The demographic characteristics of each individual were also obtained by the means of a questionnaire. This study was approved by the Ethics Committee of the Isfahan University of Medical Sciences (ref: IR.MUI.REC.1395.3.173).

The exclusion criteria for selecting healthy controls were suffering from any autoimmune disease, previous organ transplantation and suffering from any inflammatory disease according to the results of erythrocyte sedimentation rate and C-reactive protein, and multiple sclerosis. The number of patients and controls were set according to a study analyzing the expression rate of *OX40* (22) and according to the sample calculation formula by assuming 20% overexpression in patients, 95% confidence interval (CI) and 80% power, a total of 20 individuals were analyzed in each group. The groups were matched by demographic characteristics (i.e. age and sex). 

A total of 5 ml venous blood was taken from each individual by observing safety and health recommendations, of which 2 ml was collected in EDTA- containing tubes to extract RNA. The other 3 ml of blood was kept in a distinct tube without any anti-coagulant for serum analysis.

### ELISA

The blood samples collected for serum analysis were centrifuged at 4000 rpm for 6 minutes to isolate the serum. One hundred microliter of the serum sample was poured into a separate tube and stored in a freezer at -20°C. To measure the level of serum OX40, human sCD134 (OX40) Platinum ELISA-eBioscience was used and standard curve was drawn using the derived values based on data from the ELISA reader (Hiperion, Germany). Optical density (OD) at 450 nm wavelength was recorded in the samples of the two groups. The mean OD was 0.061 and 0.057 nm in the patient and control groups respectively.

### *OX40* gene expression

Total RNA was extracted using the YTA Total Purification mini kit for blood (Qiagen, China) according to the standard protocol. cDNA was synthesized with the Revertaid First cDNA synthesis kit (Fermentase, United States). 

Primers specific to *OX40* and *GAPDH*, a housekeeping gene as the internal control, were designed using the Allele ID software and BLAST (NCBI online server). The details of the primers were:

OX40 F: 5´TGGTGTAACCTCAGAAGTG3´
R: 5´GTCAACTCCAGGCTTGTA3´

GAPDH F: 5´CTCCCGCTTCGCTCTCTG3´
R: 5´TCCGTTGACTCCGACCTTC3´

In each qPCR reaction, run on a Rotor-gene 6000 (Corbett Life Science, Australia), a 10 µl mixture consisting of 6.25 ml SYBR Green Master Mix (Applied Biosystems, UK), 0.5 ml (5 pmol) of each primer, 1.75 ml nuclease free water and 1 ml (5 ng) genomic DNA. The reactions of each cDNA sample were simultaneously conducted in triplicate for each pair of genes and the mean Ct was calculated for each gene. The cycling conditions were an initial denaturation at 95°C for 5 minutes, followed by 40 cycles of 95°C for 15 seconds, 60°C for 20 seconds, and 72°C for 25 seconds. 

To investigate the outcome of amplification reactions of both genes, standard dilutions (50-3.13 ng/microL) of cDNA of each gene were prepared. After qPCR, the standard curve was drawn for each PCR fragment. PCR efficiency was calculated by determining the slope of the standard curve and using the formula efficiency=[10^-1/slope^]. Relative expression was calculated according to Schmittgen and Livak (23).

To ensure that RNA concentration is equal in different reactions, *GAPDH* was used as internal control. For each sample, real-time PCR was conducted for OX40 and *GAPDH* in triplicate under similar conditions, and a sample without cDNA considered negative control. For all reactions, melting curves were analyzed to ensure the specificity of the PCR product of interest.

### Statistical analysis

In this study, statistical analyses were performed using the Statistical Package for the Social Sciences (SPSS, IBM corporation) software version 19. The Shapiro-Wilk test was used to investigate normality of the data. The data were then analyzed by a nonparametric test, i.e. the Mann-Whitney U test. For all comparisons, P<0.05 was considered as statistically significant.

## Results

### Patients

The patient group consisted of fifteen males and five females. The mean age of the patient group was 67 (range of 46-88) years. Demographic characteristics of the of the two groups of patients and controls, and baseline characteristics, based on the PD Questionnaire-39 have listed below (Tables1, 2). The distribution of age and gender in the two groups was not significantly different (P>0.05). 

**Table 1:** Distribution of demographic characteristics in both study groups


Group		Mean ± SD	P value	Sex		P value
				Female	Male	

Age (Y)						
	Control	69.05 ± 9.43	0.805	15	5	0.642
	Patient	69.80 ± 9.66		15	5	


**Table 2:** The baseline characteristics of patients


Characteristics of patients	Mean ± SD n=18

Age of onset (Y)	65.78 ± 11.72
Disease duration	4.11 ± 3.36
PDQ-39	
D1	55.28 ± 30.29
D2	41.20 ± 27.37
D3	42.13 ± 33.15
D4	47.57 ± 43.73
D5	19.21 ± 10.31
D6	37.50 ± 27.87
D7	36.57 ± 33.60
D8	60.64 ± 23.88
Total (PDSI)	42.51 ± 20.43
Family history,	n (%)
Yes	5 (27.8)
No	13 (72.2)


PDQ; Parkinson’s Disease Questionnaire, PDSI; Parkinson’s disease symptom inventory, and D; Discrete scale.

### ELISA

**Fig.1:**
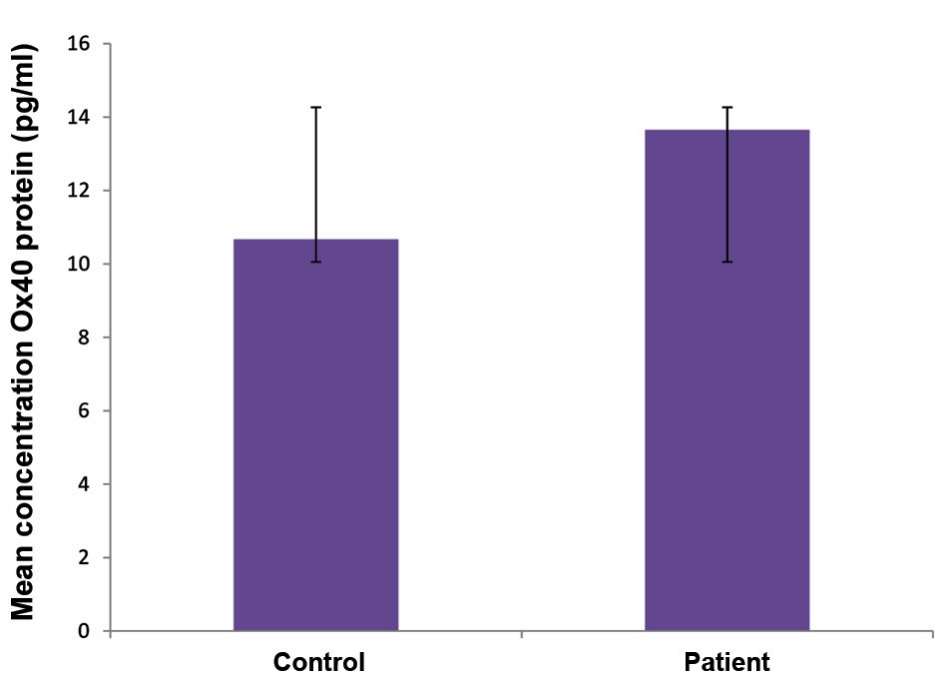
The mean serum concentration of OX40 in the patient and control groups.

According to the standard curve and OD of the studied samples, OX40 concentration of the participants was calculated. The mean serum concentration of OX40 protein was 13.65 pg/ml in the patients and 10.67 pg/ml in the controls. Given that this variable was not normally distributed in the two groups (P<0.05), the Mann-Whitney U test was used to compare the independent groups. Although the mean concentration was higher in the patient group, the difference was not significant (P=0.144, Fig .1).

### Real-time polymerase chain reaction

#### Quantity and quality of extracted RNA

Concentration of RNA at the 260 nm and 280 nm wavelengths was calculated and its ratio (260/280) was between 1.8 to 2. The quality of RNA extracted was examined by agaroz gel electrophoresis.

#### *OX40* expression analysis

**Fig.2:**
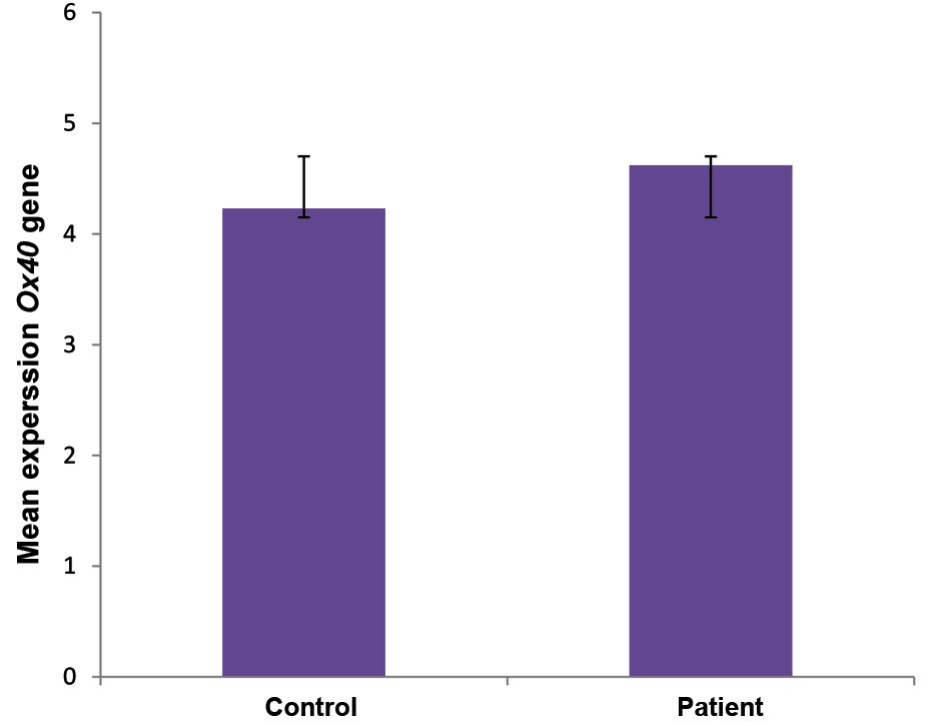
The relative expression of OX40 in peripheral blood of the patient and control groups.

As shown in Figure 2, the mean relative expression of *OX40* was 4.89 and 4.23 in the patient and control groups. Because the studied variable was not normally distributed in the two groups (P<0.05), the Mann-Whitney U test of two independent samples was used. Although *OX40* expression was increased in the patient group, the difference was not statistically significant (P=0.433, Fig .2). 

### The relationship of OX40 transcript and protein expression levels with gender

We observed no significant difference in OX40 transcript and protein expression between men and women of the patient group (P>0.05). Gender-specific expression values are listed below (Table 3).

**Table 3:** Comparison of OX40 transcript and protein expression between males and females in the patient group


	Mean ± SD	P value
	n=15	

*OX40* Gene		
Male	5.33 ± 1.91	0.434
Female	2.49 ± 2.01	
Concentration of OX40 protein		
Male	14.73 ± 2.90	0.435
Female	10.42 ± 3.06	


### The relationship of *OX40* transcript and protein expression levels with age. 

Results showed no correlation between age and *OX40* expression (P=0.506, r=0.158). Similarly, no correlation was found at the protein level (P=0.229, r=0.282). 

### Correlation between transcript and protein levels of*OX40*

**Fig.3:**
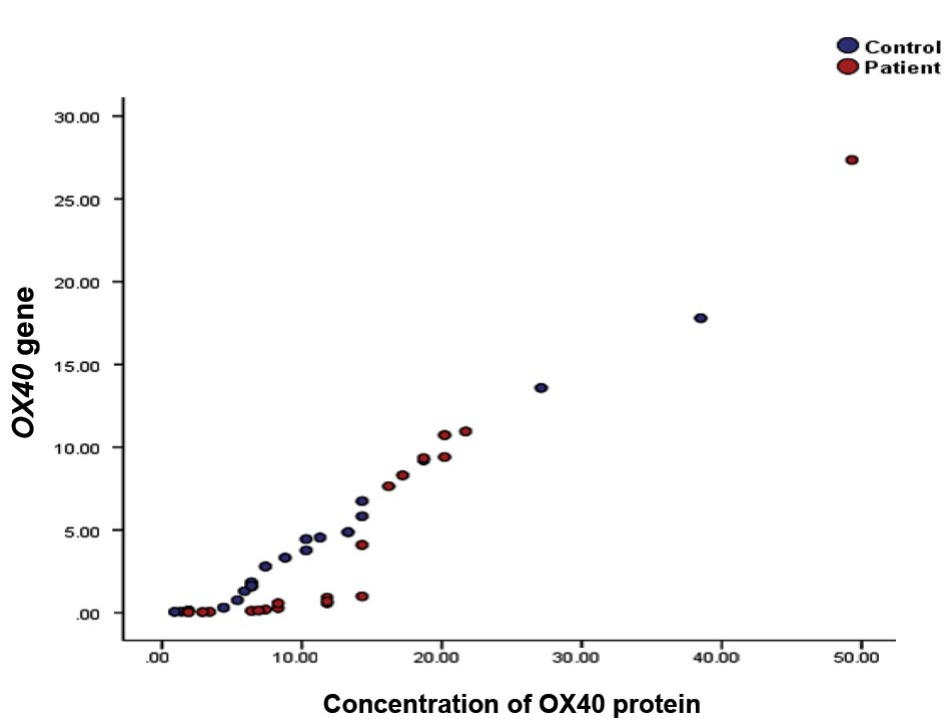
Scatter plot of patients based on their OX40 expression at the transcript (y-axis) and protein (x-axis) levels. Expression at the two levels were significantly correlated (P=0.001)

We observed a significant correlation between the transcript and protein levels of *OX40* expression (P=0.001, r=0.888, Fig .3). 

## Discussion

PD is a common disease of CNS and the motor system with underlying genetic, epigenetic, and environmental factors. Monogenic disorders and mutations in certain genes such as LRRK2, PINK1 and PARKIN may cause PD with different levels of penetrance depending on the genotype of the individual (24). However, there are other genes that may cause dopaminergic neuron damage due to certain changes such as fighting oxidative stress, apolipoprotein E (APOE), glucocerebrosidase, monoamine oxidase and microtubule-associated protein tao (MAPT), and therefore increase predisposition to develop PD (25).

In recent years, the role of other proteins in increasing predisposition to PD has been investigated, including genes involved in inflammatory processes and mechanisms of acquired and innate immunity. For instance, the association between inflammation of peripheral neuritis and nerve damage in patients with PD has been reported based on clinical evidence. Su and Federoff (26) demonstrated that systemic inflammation in PD patients included increased serum levels of TNF-a and its receptor as well as interlukin-6 (IL-6) compared with the controls. CD134 (OX40 protein) is a tumor necrosis factor receptor family. Its ligand, OX40L (CD134L), is a member of the TNF family and is expressed on activated T cells, B cells, dendritic cells, macrophages, endothelial cells and microglia. The OX40-OX40L interaction plays a key role in function and viability of activated T cells and production of T memory cells (27, 28).

We observed no significant change in *OX40* expression at either the transcript or protein level. However, it is necessary to conduct comprehensive genetic and epigenetic studies to explain the genetic factors in complex diseases such as PD. Different genetic risk factors for PD have been investigated. Few of the genetic factors are transferred in a monogenic Mendelian fashion such as the autosomal dominant mutations in genes encoding alphasynuclein (SNCA), Leucine-rich repeat kinase 2, and VPS35, and autosomal recessive mutations in PINK1/ PARKIN/DJ-1, glucocerebrosidase, PLA2G6, FBX07 and ATP13A2 (29). In addition, the role of defective mitochondrial function in PD due to mutations in the nuclear and mitochondrial genomes has been frequently investigated (30).

Medical knowledge about the genetic predisposition to certain diseases such as PD has grown remarkably in the recent decades. In fact, detecting clinical characteristics of monogenic types of PD has resulted in identifying defective pathways in its pathogenesis including defective mitochondrial function, new signaling pathways and protein metabolism. In contrast, study of large populations indicates that variants with lower risk have a 30% contribution to heritability of PD sporadic types due to high frequency. The remaining contribution to PD heritability depends on genes with more balanced risk and more recent mechanisms such as somatic mutations and epigenetic effects (31). It is, however, not still possible to predict the incidence of this disease in people, which represents the genetic complexity of this disease. Accordingly, the expression rate of a single gene and the serum level of its protein could not influenced by of the interactions.

We suggest that the lack of association in this study may be due to sample size or blood cells may not be a suitable representative for the CNS. In contrast to the present study, some studies indicate increased OX40 protein and its ligand on T cells surface helps launch inflammatory pathways of necrosis and apoptosis (28). Understanding the exact etiology of a disease depends on the careful study of its phenotype.

PD is diagnosed with clinical conditions, including asymmetrical movements, slow movements, and the response to dopamine therapy. Given the differences that are manifested in the PD phenotype spectrum, the genotype-phenotype correlation is highly complex. Except a few cases of monogenic Mendelian inheritance patterns, polygenic predisposition to PD is likely to be the genetic basis of most patients and has thus been much frequently investigated (32). For instance, Mendelian inheritance is not observed in patient generations (33) and even identical twins show disconcordance (34, 35). 

Moreover, epigenetic factors can be effective on the expression profile of the genes as well as incidence of different phenotypes. Information on epigenetic factors is still limited but studies on this area will probably help explain how certain diseases such as PD are inherited. Epigenetics make it possible to control certain processes such as methylation, phosphorylation, acetylation, and production of gene expression-regulating microRNAs in the presence of a specific stimulus. For example, according to recent studies, methylation in substantia nigra in people with sporadic PD can decrease SNCA expression (36, 37). In addition, the expression patterns of different microRNAs can lead to defective mitochondrial function in people with PD (38).

It is therefore likely that in the patient group analyzed here, the interactions among various genes as well as different environmental and epigenetic factors may have led to change in the expression profile of the studied gene. It can therefore be argued that the expression of a specific gene that is hypothetically associated with the incidence of a disease may be influenced by different regulatory pathways and environmental conditions as well as the expression of other genes.

## Conclusion

We conclude that given the lack of significant expression change of *OX40* in blood samples of PD patients, measuring the expression of this gene in blood is unlikely to be a biomarker for PD diagnosis. Additional clinical, cellular and interventional studies are therefore needed to assess its expression directly in the CNS and also develop novel biomarkers.
